# Tremor Asymmetry and the Development of Bilateral Phase‐Specific Deep Brain Stimulation for Postural Tremor

**DOI:** 10.1002/mds.30275

**Published:** 2025-06-23

**Authors:** Shenghong He, Alceste Deli, Timothy O. West, Fernando R. Plazas, Alek Pogosyan, Christoph Wiest, Laura Wehmeyer, Fahd Baig, Francesca Morgante, Pablo Andrade, Michael G. Hart, James J. FitzGerald, Veerle Visser‐Vandewalle, Erlick A. Pereira, Alexander L. Green, Huiling Tan, Hayriye Cagnan

**Affiliations:** ^1^ Medical Research Council Brain Network Dynamics Unit, Nuffield Department of Clinical Neurosciences University of Oxford Oxford UK; ^2^ Nuffield Department of Surgical Sciences University of Oxford Oxford UK; ^3^ Department of Bioengineering Imperial College London London UK; ^4^ Department of Stereotactic and Functional Neurosurgery, University Hospital Cologne, and Faculty of Medicine University of Cologne Cologne Germany; ^5^ Neurosciences Research Centre, Molecular and Clinical Sciences Institute, City St. George's University of London London UK; ^6^ Nuffield Department of Clinical Neurosciences University of Oxford Oxford UK

**Keywords:** postural tremor, phase‐locked deep brain stimulation, asymmetry, instability

## Abstract

**Background:**

Tremor phase‐locked deep brain stimulation (DBS) has been shown to modulate symptom severity in postural tremor, including essential and dystonic tremor, with less energy than existing systems. Previous studies focused on unilateral stimulation; it remains unknown how tremor asymmetry interacts with stimulation in the context of bilateral phase‐locked DBS.

**Methods:**

Archival limb acceleration from nine essential tremor patients was analyzed for asymmetries in tremor amplitude, frequency, and instability, and their relationship with continuous high‐frequency DBS (cDBS). Bilateral phase‐locked DBS was tested in one essential tremor and one dystonic tremor patient.

**Results:**

Postural tremor is asymmetric, with larger tremor power linked to smaller amplitude and frequency instability in one hand. These asymmetries were significantly reduced during cDBS, with greater effects on larger amplitude tremors. Bilateral phasic DBS effects were also asymmetric.

**Conclusions:**

This study enhances understanding of tremor asymmetry and its relationship with DBS, offering insights for patient‐specific tremor treatments. © 2025 The Author(s). *Movement Disorders* published by Wiley Periodicals LLC on behalf of International Parkinson and Movement Disorder Society.

Tremor is one of the most common symptoms in movement disorders.^1^ Deep brain stimulation (DBS) targeting the ventral intermediate nucleus (VIM) of the thalamus is a standard treatment for medication refractory tremors.^2^ However, continuous high‐frequency DBS (cDBS) may disrupt not only pathological neural signals driving patients' symptoms, but also physiological neural activities giving rise to stimulation‐induced side effects such as impairments in speech, balance, and gait, particularly when the patients adapt to the stimulation over time and thus higher stimulation intensities are required.^2^, ^3^ Previous studies demonstrated that significant tremor relief can be achieved in selected patients during unilateral DBS, phase‐locked to tremor from the contralateral hand.^4^, ^5^ Considering that most tremor patients have bilateral tremor,^6^ it remains unknown how postural tremor asymmetry interacts with bilateral phase‐locked DBS. In this study, we compared multiple tremor characteristics across both hands in no DBS and cDBS conditions in nine patients with essential tremor. In addition, we piloted bilateral, tremor phase‐locked DBS in two tremor patients (one essential and one dystonic tremor).

## 1 Methods

Eleven patients (adults, four females) with postural tremor participated in this study (patients P1–P6 were published previously,^7^ P10 was diagnosed with dystonic tremor, while all other participants were diagnosed with essential tremor). All participants underwent bilateral implantations of DBS electrodes targeting the VIM thalamus and/or posterior subthalamic area (Fig. S1).^8^ The study was approved by the local ethics committees and all patients provided their informed written consent according to the Declaration of Helsinki. Recordings from nine patients during cDBS were conducted acutely during lead externalization (3–5 days after lead implantation), with the stimulation parameters optimized on site as described in the Supplementary Methods and our previous study.^9^ Each participant was asked to maintain a tremor‐provoking posture such as raising both arms to shoulder level with flexed elbows and the fingers of both hands pointing to the center while sitting. The task was repeated in 6–8 blocks. Each block involved holding the tremor‐provoking posture for approximately 30 s, followed by 30‐s rest. Tremor phase‐locked DBS experiments (n = 2) were conducted after internalization of the leads and implantable pulse generators (IPGs) for 1.5 months (P10) and 4 years (P11), respectively, using each patient's clinically optimized stimulation parameters. In this study, Nexus‐D4 (Medtronic, Minneapolis, MN, USA), an investigational device, was used to deliver phase‐locked bilateral stimulation (Fig. 2A). Specifically, we first tracked instantaneous tremor phase in one hand, for example, the right hand, using previously described methods,^4^, ^5^ and used Nexus‐D4 to deliver stimulation to first the left VIM followed by a burst of stimulation to the right VIM (Fig. 2B). Subsequently, we used tremor phase derived from the other hand (ie, the left hand) to control stimulation delivered to both hemispheres. More details can be seen in Supplementary Methods. This allowed us to investigate the effect of tracking tremor phase from one hand to control stimulation timing bilaterally and the effect of tracking tremor phase from both hands through post‐hoc analysis. Note that, ideally, we would need two separate IPGs to independently control the timing of the stimulation to each hemisphere (phase‐locked to the contralateral tremor), which is not possible in existing chronic DBS patients.

**FIG. 1:**
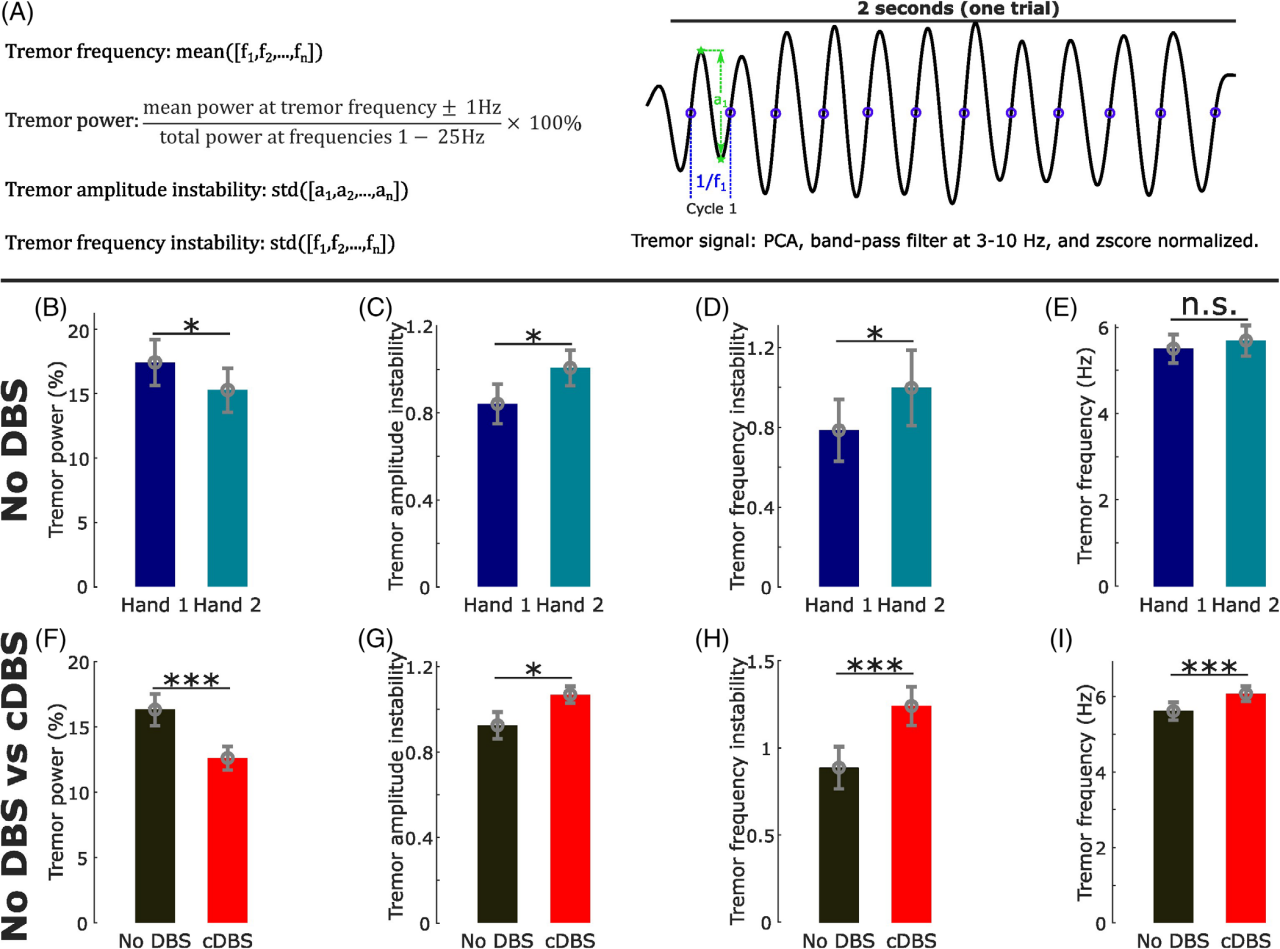
Postural tremor was asymmetric in terms of power and instability. These asymmetric measurements were modulated differently by continuous high‐frequency deep brain stimulation (cDBS) on different hands. (A) A demonstration of the quantifications of tremor frequency, power, amplitude instability, and frequency instability. (B–E) Comparisons of tremor power (B), amplitude instability (C), frequency instability (D), and frequency (E) between tremor predominant (Hand 1) and non‐dominant (Hand 2) hands measured during no DBS condition. (F–I) Comparisons of tremor power (F), amplitude instability (G), frequency instability (H), and frequency (I) between no DBS and cDBS conditions. [Color figure can be viewed at wileyonlinelibrary.com]

**FIG. 2:**
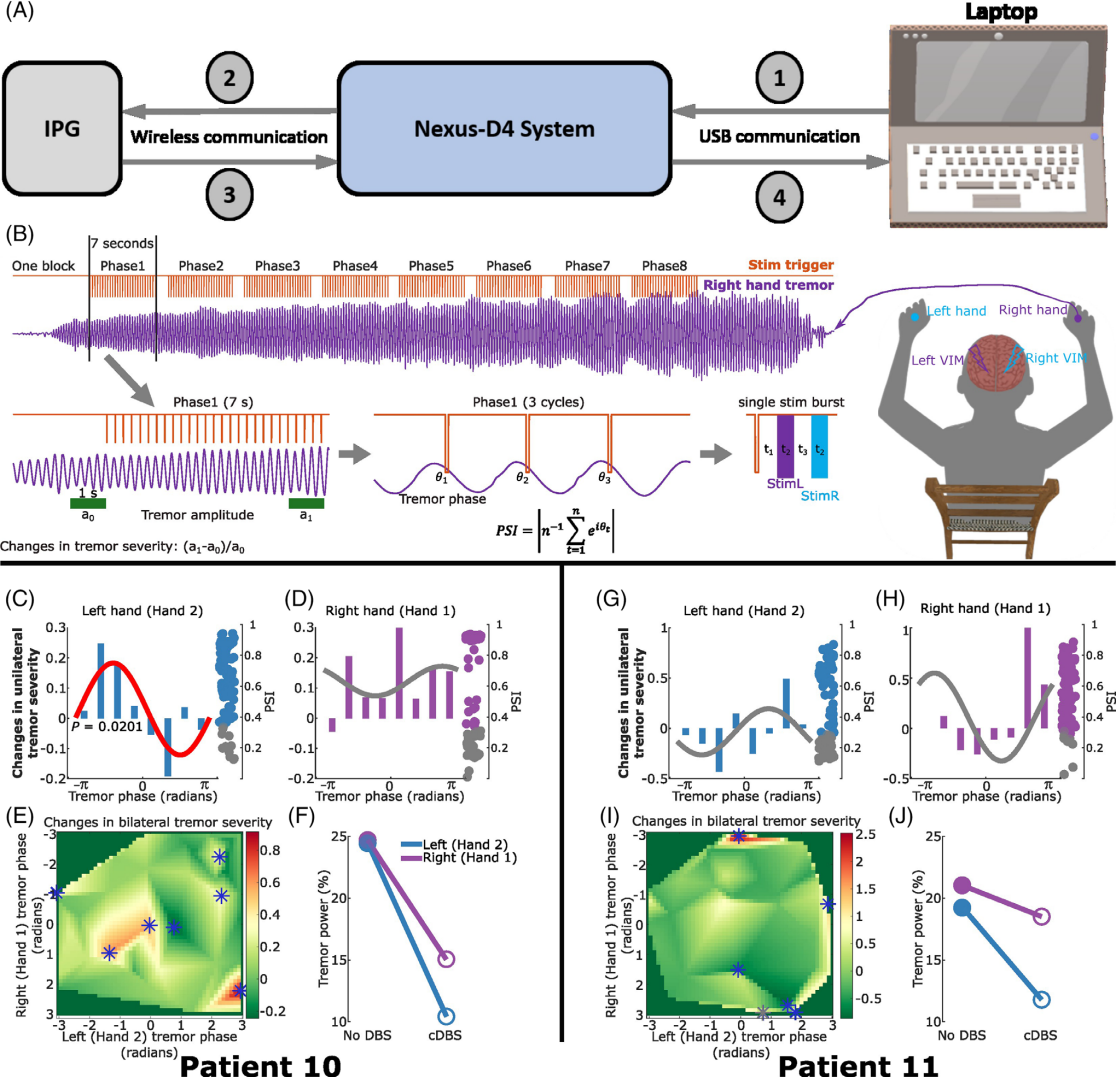
Protocol of tremor phase‐locked deep brain stimulation (DBS) and its effect on tremor severity. (A) A schematic of the communication loop between host laptop and patient's implantable pulse generators (IPGs) via the Nexus‐D4 system. (B) A demonstration of right‐hand tremor phase‐locked bilateral stimulation. Tremor signals are recorded using a triaxial accelerometer. The dominant tremor axis is determined and real‐time filtered at the patient‐specific tremor frequency band, as shown in purple. Stimulation is delivered in blocks, with each block containing eight trials. In each trial, stimulation is triggered at one of the eight predefined tremor phases for 7 s, as shown in orange. Within each trial, one trigger is sent out at a specific tremor phase. Following each trigger, two bursts of stimulation with a fixed interval of 71.48 ± 3.18 ms (t_3_) were delivered to the contralateral followed by the ipsilateral ventral intermediate nucleus (VIM) relative to the hand used for tremor phase tracking. A fixed time interval of 109.05 ± 2.42 ms (t_1_) was induced by the Nexus‐D4 between each trigger and the first burst of stimulation. The duration of each burst of stimulation was about 35 ms (t_2_). Stimulation effect is evaluated by comparing the tremor amplitude (on one or both hands) in the last second within each trial relative to 1 s immediately before the start of each trial, that is, changes in tremor severity. The accuracy of phase‐locking is evaluated by quantifying the phase synchrony index (PSI) across all stimulation triggers within each trial. (C, D) Effects of bilateral tremor phase‐locked stimulation on one hand. Each bar indicates the effect of a specific tremor phase bin, with the curve representing the fitted sine curve considering all bins. Each dot on the right side indicates accuracy of the corresponding phase‐locked stimulation trial, quantified using PSI. The red sine curve indicates significance against the surrogate distribution after correcting for multiple comparisons. Grey dots for PSI indicate non‐significant phase‐locking trials against surrogate distributions, which have been excluded from the analysis. (E) Effects of bilateral tremor phase‐locked stimulation on both hands. Bilateral phasic stimulation effect on tremor severity measured from both hands. Blue stars indicate marginally significant phasic effects against surrogate distribution, which did not survive multiple comparison corrections though. (F) Effects of continuous high‐frequency DBS (cDBS) on tremor severity for left (blue) and right (purple) hands. (G–J) The same as (C–F) but for Patient 11. Note that the phases shown in this figure should be interpreted as the peripheral tremor phase shifted by 109.05 ± 2.42 ms, caused by the delay between the command sent to Nexus‐D4 and the execution of the command by the IPG. Using the peripheral phase assumes that the mapping between peripheral tremor and the central neural activity driving it is static. However, based on previous research,^5^ it is plausible that this relationship changes dynamically across different tasks. [Color figure can be viewed at wileyonlinelibrary.com]

To investigate tremor asymmetry, we (1) segmented limb acceleration measurements into 2‐s non‐overlapping trials, (2) quantified tremor characteristics including frequency, power, and cycle‐by‐cycle instability defined by the standard deviations of amplitude and frequency across tremor cycles for each 2‐s trial (Supplementary Methods), and (3) compared them between hands in both no DBS and cDBS conditions, as illustrated in Figure 1A. Here trials with tremor power less than 7.5% (ie, 1.5 times the mean normalized power in the frequency band of 1–20 Hz) were excluded. In this study, asymmetric tremor was defined as a condition in which there were significant differences in the aforementioned tremor characteristics between hands. To investigate the effect of tremor phase‐locked stimulation, for each phasic stimulation trial (here one trial represents tracking a specific tremor phase from one of the limbs for 7 s), we quantified the change in tremor severity in one hand or both hands by comparing the tremor amplitude between the last 1 s of phasic stimulation and the last 1 s before stimulation onset at that specific phase (Fig. 2B), similar to the method used in previous studies.^4^, ^5^ Phasic stimulation was delivered in blocks, with each block containing eight trials to track eight distinct tremor phases, resulting in a phase resolution of 45°. In total, five blocks of phasic‐DBS were conducted for each limb of each patient. A similar amount of tremor data were also recorded during cDBS and no DBS conditions. The order of experimental conditions (cDBS, no DBS, and phasic‐DBS) was randomized for each patient, and a brief break of ~5 min was included after turning off the DBS before starting the no DBS experiment. See Supplementary Methods for more details on cDBS, tremor phase‐locked DBS, and data recording. Clinical and stimulation details of all patients are summarized in Table S1.

Statistical analyses were conducted using custom‐written scripts in MATLAB R2021‐b (The MathWorks Inc., Nantucket, MA, USA). Tremor characteristics were quantified on individual trial basis (including frequency, power, amplitude instability, and frequency instability), and a generalized linear mixed effect modelling was used to investigate the difference between hands, stimulation conditions, as well as the interaction between the two.^10^ Multiple comparisons applied to these measurements were corrected using the false discovery rate (FDR) approach.^11^, ^12^ The estimated value with standard error of the coefficient (*k* ± SE), pre‐corrected *P*‐values as well as their significances after FDR correction were reported. To test the effects of bilateral phasic stimulation, we tested the significance of the change in tremor severity for each phase bin against a surrogate distribution (D_1_) with 1,000,000 points representing the natural tremor variability without DBS. Specifically, we randomly selected 50,000 7‐s segments of tremor during no DBS and quantified the natural changes in tremor severity to generate a surrogate distribution D_0_. For each phase bin, we randomly selected N values from D_0_ and quantified their median value (with N corresponding to the number of stimulation trials delivered at this phase bin). This was repeated 1,000,000 times, leading to the surrogate distribution D_1_.^4^, ^5^ To investigate the overall tremor modulation and different modulation patterns across hands, we fitted a sine curve to the changes in tremor severity over all tested tremor phase bins for each hand. The amplitude of the fitted sine curve was tested against a surrogate distribution (D_2_) with 1,000,000 points in which each point indicated the amplitude of the fitted sine curve derived from the surrogate distribution D_1_ for the same hand.^13^ To control for the accuracy of phase‐locked stimulation, we quantified the phase synchrony index (PSI) across tremor phases, at which stimulation was delivered in each trial, and tested this against a surrogate distribution with 50,000 points, with each point indicating the PSI quantified after shuffling the tremor phase relative to the stimulation timing.

## 2 Results

### 2.1 Postural Tremor was Asymmetric Across Hands

In the absence of stimulation, on average, the tremor‐dominant hand (‘Hand 1’) defined as the hand with larger tremor power (Fig. 1B, *k* = −2.3239 ± 0.9646, *P* = 0.0161), had significantly less tremor instability in terms of amplitude (Fig. 1C, *k* = 0.1371 ± 0.0655, *P* = 0.0364) and frequency (Fig. 1D, *k* = 0.2551 ± 0.1075, *P* = 0.0177) when compared with the non‐tremor‐dominant hand (‘Hand 2’), although the peak tremor frequencies were similar between the hands (Fig. 1E). These differences were no longer significant during cDBS (Table S2), that is, cDBS reduced tremor asymmetries. Overall, apart from reducing tremor power by about 23% (Fig. 1F, *k* = −3.7289 ± 0.9367, *P =* 7.0595 × 10^−5^), cDBS significantly increased tremor instability (amplitude: Fig. 1G, *k* = 0.1754 ± 0.0659, *P =* 0.0078; frequency: Fig. 1H, *k* = 0.3836 ± 0.0962, *P* = 6.8512 × 10^−5^) and peak tremor frequency (Fig. 1I, *k* = 0.5277 ± 0.1030, *P =* 3.2617 × 10^−7^). An interaction analysis revealed that the tremor asymmetries, that is, the differences in the tremor characteristics between Hand 1 and Hand 2, were significantly reduced by cDBS (except for frequency instability), and the effects of cDBS on these tremor characteristics were significantly stronger on Hand 1 compared with Hand 2 (except for frequency instability). A separate interaction analysis revealed no significant difference in the tremor asymmetry between the included acute and chronic patients (Table S2).

### 2.2 Phasic DBS had Different Effects on Different Hands

For both patients and hemispheres, while considering the changes in tremor severity on one hand only, when bilateral phasic stimulation was delivered to the contralateral VIM first, there was no distinct phase bin that showed significant modulation compared with the natural tremor variability (see the statistical analysis section in Methods for more details). However, the overall stimulation effect was significant in P10, left hand (Fig. 2C, *P* = 0.0201 against surrogate distribution) when using a fitted sine curve to test significance and using FDR to correct multiple comparisons. This was also reflected during cDBS: the most prominent effect was seen in P10 left hand (Fig. 2F,J). Tremor modulation profiles during phasic DBS (Fig. 2C,D,G,H) and the modulation of other tremor characteristics with cDBS (Fig. S2) were different across hands, analogous to the results highlighted in the previous Results section. While considering the changes in tremor severity for both left and right hands (by taking the average change in tremor severity) for a given tremor phase combination derived from instantaneous tremor phases from both hands, both patients showed multiple phase combinations that provided marginally significant effects on bilateral tremor severity (Fig. 2E,I, blue stars, *P* < 0.05 against surrogate distribution, although none of them survived multiple comparison corrections). These results suggest that phase‐locked DBS delivered to the two hemispheres targeting the optimal tremor phases should be determined for left‐ and right‐hand jointly.

## 3 Discussion

To limit the emergence of stimulation‐induced side effects, different closed‐loop DBS protocols have been proposed, including switching on DBS only when tremor or a tremor‐provoking movement is detected^7^, ^14^, ^15^ or delivering stimulation time‐locked to a specific tremor rhythm.^4^, ^5^ The first strategy is based on the intermittent nature of postural essential and dystonic tremor, while the latter approach aims to specifically disrupt the underlying neural activity related to tremor. In a recent study involving a clinical survey of 487 individuals diagnosed with essential tremor, Whaley et al. reported that about half (52%) of the cohort reported bilateral tremor onset, and about 90% of the individuals eventually presented bilateral tremor.^6^ A separate study found that while bilateral VIM DBS provided greater overall tremor reduction across both sides compared with unilateral DBS, unilateral stimulation was just as effective in alleviating tremors in the contralateral hand.^16^ Here, we showed that postural tremor is asymmetric in terms of cycle‐by‐cycle tremor frequency, power, and instability (amplitude and frequency) in the absence of DBS as well as when bilateral cDBS was on. Effects of phase‐locked DBS on different hands were also asymmetric. Results from a separate study also showed that the efferent thalamic to tremor connectivity was lateralized in essential tremor, with a stronger connectivity from the contralateral VIM, which was associated with bigger and more stable tremor as well as larger cDBS effects. In addition, more unstable tremor was associated with stronger cross‐hemisphere coupling between left and right thalami.^9^ These results taken together suggest that tremor in different hands might be associated with different but interacting oscillatory sources. Bilateral phase‐locked DBS independently targeting the optimal suppressive phases for left‐ and right‐hand tremor might work better in disrupting the relevant oscillatory sources, and thus be more effective at suppressing pathological tremors, whist simultaneously reducing the total electrical energy delivered.

There are several limitations to this study. First, we observed that tremor in the non–tremor‐dominant hand (Hand 2) was more unstable, but it is unclear whether this instability was due to other clinical features, such as ataxia. Second, this study focused solely on postural tremor; it remains unclear how the results would generalize to other activities such as writing. Third, the setup used for phasic DBS in this study was designed as a proof‐of‐concept and, in future clinical applications, should leverage depth signals to minimize the instrumentation required for implementing phasic control.

## Author Roles

(1) Research Project: A. Conception, B. Organization, C. Execution; (2) Statistical Analysis: A. Design, B. Execution, C. Review and Critique; (3) Manuscript Preparation: A. Writing of the First Draft, B. Review and Critique.

S.H.: 1A, 1B, 1C, 2A, 2B, 2C, 3A, 3B.

A.D.: 1B, 1C, 3B.

T.O.W.: 1A, 1B, 2A, 2C, 3B.

F.R.P.: 1C, 3B.

A.P.: 1A, 1B, 3B.

C.W.: 1C, 2C, 3B.

L.W.: 1C, 3B.

F.B.: 1C, 2C, 3B.

F.M.: 1B, 3B.

P.A.: 1B, 3B.

M.G.H.: 1B, 3B.

J.J.F.: 2C, 3B.

V.V.‐V.: 1B, 3B.

E.A.P.: 1B, 3B.

A.L.G.: 1A, 1B, 2C, 3B.

H.T.: 1A, 1B, 2C, 3B.

H.C.: 1A, 1B, 2C, 3B.

## Financial Disclosures of All Authors (for the Previous 12 Months)

S.H., F.R.P., A.P., C.W., L.W., and H.T were supported by the Medical Research Council (MRC) (MC_UU_00003/2), the Medical and Life Sciences Translational Fund (MLSTF) from the University of Oxford, the National Institute for Health and Care Research (NIHR) Oxford Biomedical Research Centre, and the Rosetrees Trust, UK. S.H. is supported by a Non‐Clinical Postdoctoral Fellowship from the Guarantors of Brain and an International Exchanges Award (IES\R3\213,123) from The Royal Society. A.D. was supported by the Alexander S. Onassis Public Benefit Foundation (F ZK 085_2). T.O.W and H.C. were supported by the MRC (MR/R020418/1 and MR/X023141/1). A.G. was supported by the MRC (MC_PC_16056). E.A.P. has received speaking honoraria from Boston Scientific and research support from NIHR, UK Research and Innovation (UKRI), Life after Paralysis, and Rosetrees Trust. F.M. has received speaking honoraria from AbbVie, Medtronic, Boston Scientific, Bial, and Merz; travel grants from the International Parkinson's Disease and Movement Disorder Society; advisory board fees from AbbVie, Merz, and Boston Scientific; consultancy fees from Boston Scientific, Merz, and Bial; research support from NIHR, UKRI, Boston Scientific, Merz, and Global Kynetic; royalties for the book *Disorders of Movement* from Springer and is a member of the editorial boards of *Movement Disorders, Movement Disorders Clinical Practice*, and the *European Journal of Neurology*. M.G.H. is a member of the Medicines and Healthcare products Regulatory Agency (MHRA) Interim Devices Working Group. V.V.‐V. is a member of advisory boards and has received speaker's honoraria from Medtronic, Boston Scientific, and Abbott. J.J.F. reports advisory board, consulting fees, and educational honoraria from Abbott, consultancy for Eli Lilly, and research grants from UCB and Merck. F.B. and P.A. report no disclosures. There are no other relationships or activities that could appear to have influenced the submitted work.

## Supporting information


**Data S1.** Supporting Information.

## Data Availability

The scripts and some raw data required to reproduce the analyses in this article have been published under a CC BY‐SA license: https://doi.org/10.60964/bndu-1tv5-4967. The remaining raw data are available by contacting Dr Hayriye Cagnan (h.cagnan@imperial.ac.uk) and would potentially be subject to a Data Transfer Agreement.
