# Supplementary material for: Tremor Asymmetry and the Development of Bilateral Phase‐Specific Deep Brain Stimulation for Postural Tremor
Source: Mov Disord. 2025 Jun 23;40(9):1977–82. doi: 10.1002/mds.30275 (PMC12485572; doi:10.1002/mds.30275)
Supplement: Supplementary file 1 — Data S1. Supporting Information. [file MDS-40-1977-s001.docx]

**Supplementary materials**

**Methods**

**Tremor characteristics**

For preprocessing, the recorded continuous acceleration measurements were band-pass filtered at 1–20 Hz using a fourth order zero-phase Butterworth IIR digital filter in MATLAB (R2023-b, MathWorks). After filtering, a principal component analysis (PCA) was applied on the tri-axial acceleration measurements, and the first component was selected as the measurement of tremor on a given hand. After preprocessing, we split the data during tremor-provoking posture holding into non-overlapping 2 s segments and considered each segment as a trial. For each trial, power spectral density (PSD) was estimated using Welch’s overlapped segment averaging estimator, in a frequency range of 1–20 Hz with a 0.5 Hz resolution. On another hand, for each trial, the acceleration measurements were high-pass and low-pass filtered at 3 Hz and 10 Hz using two sixth-order zero-phase Butterworth IIR digital filters, followed by z-score normalisation. Then, zero-crossing points from negative to positive were used to identify the individual tremor cycle within each trial. For each tremor cycle, the instantaneous tremor amplitude was quantified as the distance between the peak and trough, while instantaneous tremor frequency was defined as the reciprocal of the duration of the tremor cycle, as shown in Fig. 1A. Finally, tremor frequency was identified as the mean of the instantaneous cycle-by-cycle tremor frequencies. Tremor power was quantified as the mean of the normalized power at tremor frequency ± 1 Hz. Tremor amplitude and frequency instability were quantified as the standard deviation of the instantaneous tremor amplitude and frequency across cycles, respectively (He et al., 2024).

**Continuous high-frequency DBS**

Recordings from nine patients were conducted 3 to 5 days after the electrode implantation, when the DBS leads were temporarily externalized, and a configurable neurostimulator was used to deliver cDBS. For cases with implanted directional leads, segmented contacts were used in the ring mode. The stimulation contact was selected based on: 1) imaging data and 2) a contact searching procedure to select the final stimulation contact for each hemisphere. To this end, we delivered cDBS initially at 0.5 mA, then progressively increased the amplitude in 0.5 mA increments, until clinical benefit was observed without side-effects such as paraesthesia, or until 3.5 mA was reached as the maximum amplitude (He et al., 2021; He et al., 2024).

**Tremor phase-locked DBS**

For the tremor phase-locked DBS experiments, when a desired tremor phase was detected from one of the hands, a command was sent from a laptop to the IPG through Nexus-D4, resulting in a brief burst of stimulation (about 35 ms) to be delivered to the contralateral VIM (time delay between each TTL and stimulation onset was 109.1 ± 2.5 ms, which was mainly induced by the wireless communication between Nexus-D4 and IPG), at their existing clinical settings (e.g., frequency, amplitude, contact, etc.). Subsequently, a brief burst of stimulation was delivered to the ipsilateral hemisphere with a fixed interval of 71.3 ± 3.2 ms.

**Nexus-D System**

The Nexus-D System is an investigational telemetry system for research. It is used in vitro, serving as a communication link between a host device, such as a laptop, and a Medtronic Activa family neurostimulator (i.e., IPG) of Medtronic, as shown in Fig. 2A. In our phase-locked DBS experiment, we implemented a zero-crossing algorithm in a CED Power1401-3A data acquisition interface to detect tremor phase in real time. Upon detecting the desired tremor phase, a transistor-transistor logic (TTL) pulse was sent out from the 1401 device and received by our laptop program. This triggered a command sent to Nexus-D4 via an Application Programming Interface (API). The command was then relayed to the IPG to deliver bursting stimulation.

**Data recording**

Participants were asked to maintain a tremor-provoking posture such as raising both arms to shoulder level with flexed elbows and the fingers of both hands pointing to the centre, while, limb acceleration was acquired using triaxial accelerometers taped to the back of both hands, and simultaneously recorded using a TMSi Porti amplifier (TMS International) at a sampling rate of 2048 Hz (P1-P9) or using a CED Power1401-3A data acquisition interface (Cambridge Electronic Design, Cambridge, UK) at a sampling rate of 10 KHz (P10-P11).

**Supplementary Table I: Patients details.**

| P | Age (yr) | | DD (yr) | DBS lead | | L/R Amp (mA) | | | Centre | | DBS Targeting | | Diagnose | | Predominant symptom(s) before surgery | Pre-Op Medication | |  |
| --- | --- | --- | --- | --- | --- | --- | --- | --- | --- | --- | --- | --- | --- | --- | --- | --- | --- | --- |
| 1 | 71-75 | | 18 | Abb | | 2.5 / 2.0 | | | SGH | | VIM+PSA | | ET | | Tremor, upper limb, lower limb and head tremor | None for tremor, previously tried Primidone, Clonazepam, Propranolol, Gabapentin, Topiramate | |  |
| 2 | 66-70 | | 8 | Abb | | 1.8 / 1.8 | | | SGH | | VIM+PSA | | ET | | Tremor, upper limb, with right worse than left, lower limb tremor | None for tremor, previously tried propranolol, gabapentin, topiramate, lamotrigine, primidone | |  |
| 3 | 61-65 | | 45 | Abb | | 2 / 2 | | | SGH | | VIM+PSA | | ET | | Tremor, upper limb tremor left worse than right, voice tremor | None for tremor, previously propranolol, pregabalin, primidone | |  |
| 4 | 66-70 | | 5 | Abb | | 3 / 3 | | | SGH | | VIM+PSA | | ET | | Tremor, upper limb left worse than right | None for tremor, previously Pregabalin, Primidone, Propranolol, Topiramate, Gabapentin | |  |
| 5 | 66-70 | | 47 | Abb | | 1.5 / 1.5 | | | SGH | | VIM+PSA | | ET | | Tremor, upper limb right worse than left, head tremor | None for tremor, previously tried Propranolol. Topiramate, Gabapentin | |  |
| 6 | 61-65 | | NA | Bos | | 1.1 / 1.5 | | | OUH | | VIM+PSA | | ET | | Tremor, upper limb, worse intention tremor on left | None for tremor | |  |
| 7 | 76-80 | | 20 | Bos | | 2.0 / 1.5 | | | SGH | | VIM+PSA | | ET | | Upper limbs tremor (right > left) | Propanolol, topiramate, and primidone | |  |
| 8 | 71-75 | | 15 | Abb | | 1.0 / 1.0 | | | SGH | | VIM+PSA | | ET | | Upper limbs tremor (right > left) | Propanolol and primidone | |  |
| 9 | 71-75 | | 10 | Med_1_ | | 3.5 / 1.2 | | | UHC | | VIM/PSA | | ET | | Tremor in both hands ( R>L), head tremor | previously propranolol, mylepsinum und gabapentin | |  |
| 10 | 56-60 | | 55 | Med_2_ | | 1.0 / 2.2 | | | OUH | | VIM | | DT | | Tremor upper limb. Right worse than left. Also has jaw and head tremor | Primidone 250mg tds, propranolol 40mg OD. Also on Carbamazepine 10omg for trigeminal neuralgia and pregabalin 150mg for pain | |  |
| 11 | 76-80 | | 35 | Med_2_ | | 1.7 / 3.3 | | | OUH | | VIM | | ET | | Tremor upper limbs equal bilaterally with voice and jaw tremor | None | |  |
| Mean  - | 70 | 25.8 | | |  | | 1.91 |  | |  | |  | |  | | |  | |
| SD | 5.77 | 17.22 | | |  | | 0.73 |  | |  | |  | |  | | |  | |

P = patient; yr = year; DD = disease duration; Abb = Abbott infinity 1.5mm spaced leads (1-4), Abbott; Bos = Boston linear 8 contact leads (1-8), Boston Scientific; Med_1_ = Medtronic SenSightB33015 directional leads; Med_2_ = Medtronic 3389 leads; L = left; R = right; Amp = amplitude; SGH = St George’s Hospital; UHC = University Hospital Cologne; OUH = Oxford University Hospital; VIM = ventral intermediate thalamus; ET = essential tremor; DT = dystonic tremor; SD = standard deviation. NA: Not available. Patient 1 had gait ataxia which is sometimes seen in advanced ET. Patient 2 had an overlap between ET and dystonic tremor. Patient 11 was diagnosed and received DBS for ET, but later also developed symptoms of Parkinson’s disease. Figure 1 and 2 were generated from P1-P11 and P10-P11 respectively.

**Supplementary Table II.** Comparisons on tremor characteristics between hands as well as between stimulation conditions using generalized linear mixed effect (GLME) modelling

| **Comparisons between hand1 and hand2** | | | | | | | | | | |
| --- | --- | --- | --- | --- | --- | --- | --- | --- | --- | --- |
| No DBS (Exclude, stimID==1) | | | | | cDBS (Exclude, stimID==0) | | | | | |
| **Model 1**: treFre ~ 1 + $k_{1}h\mathrm{andID}$ + 1\|subID + (-1 + handID\|subID) | | | | | **Model 1**: treFre ~ 1 + $k_{1}h\mathrm{andID}$ + 1\|subID + (-1 + handID\|subID) | | | | | |
| AIC | $k_{1}$ | $p_{1}$ | $R^{2}$ | | AIC | $k_{1}$ | $p_{1}$ | $R^{2}$ | | |
| 2343 | 0.2467 ± 0.2002 | 0.2179 | 0.8124 | | 1815.9 | -0.0090 ± 0.2074 | 0.9655 | 0.6220 | | |
| **Model 2**: trePow ~ 1 + $k_{1}h\mathrm{andID}$ + 1\|subID + (-1 + handID\|subID) | | | | | **Model 2**: trePow ~ 1 + $k_{1}h\mathrm{andID}$ + 1\|subID + (-1 + handID\|subID) | | | | | |
| AIC | $k_{1}$ | $p_{1}$ | $R^{2}$ | | AIC | $k_{1}$ | $p_{1}$ | $R^{2}$ | | |
| 8360.6 | -2.3239 ± 0.9646 | **0.0161** | 0.5868 | | 5394.1 | -1.5362 ± 1.3989 | 0.2724 | 0.5036 | | |
| **Model 3**: treAmpInst ~ 1 + $k_{1}h\mathrm{andID}$ + 1\|subID + (-1 + handID\|subID) | | | | | **Model 3**: treAmpInst ~ 1 + $k_{1}h\mathrm{andID}$ + 1\|subID + (-1 + handID\|subID) | | | | | |
| AIC | $k_{1}$ | $p_{1}$ | $R^{2}$ | | AIC | $k_{1}$ | $p_{1}$ | $R^{2}$ | | |
| 1278 | 0.1371 ± 0.0655 | **0.0364** | 0.3329 | | 685.83 | 0.0537 ± 0.0721 | 0.4567 | 0.2330 | | |
| **Model 4**: treFreInst ~ 1 + $k_{1}h\mathrm{andID}$ + 1\|subID + (-1 + handID\|subID) | | | | | **Model 4**: treFreInst ~ 1 + $k_{1}h\mathrm{andID}$ + 1\|subID + (-1 + handID\|subID) | | | | | |
| AIC | $k_{1}$ | $p_{1}$ | $R^{2}$ | | AIC | $k_{1}$ | $p_{1}$ | $R^{2}$ | | |
| 2044 | 0.2551 ± 0.1075 | **0.0177** | 0.4669 | | 1621.4 | 0.0897 ± 0.1658 | 0.5887 | 0.3670 | | |
| **Comparisons between no DBS and cDBS** | | | | | |  |  |  |  |  |
| **Model 1**: treFre ~ 1 + $k_{1}\mathrm{stimID}$ + 1\|handID + (-1 + stimID\|handID) | | | | | | | | | | |
| AIC | $k_{1}$ | $p_{1}$ | $R^{2}$ |  | |  |  |  | |  |
| 4176.1 | 0.5277 ± 0.1030 | **3.2617 × 10^-7^** | 0.7640 |  | |  |  |  | |  |
| **Model 2**: trePow ~ 1 + $k_{1}\mathrm{stimID}$ + 1\|handID + (-1 + stimID\|handID) | | | | | | | | | | |
| AIC | $k_{1}$ | $p_{1}$ | $R^{2}$ |  | |  |  |  | |  |
| 13764 | -3.7289 ± 0.9367 | **7.0595 × 10^-5^** | 0.5840 |  | |  |  |  | |  |
| **Model 3**: treAmpInst ~ 1 + $k_{1}\mathrm{stimID}$ + 1\|handID + (-1 + stimID\|handID) | | | | | | | | | | |
| AIC | $k_{1}$ | $p_{1}$ | $R^{2}$ |  | |  |  |  | |  |
| 1975.4 | 0.1754 ± 0.0659 | **0.0078** | 0.3283 |  | |  |  |  | |  |
| **Model 4**: treFreInst ~ 1 + $k_{1}\mathrm{stimID}$ + 1\|handID + (-1 + stimID\|handID) | | | | | | | | | | |
| AIC | $k_{1}$ | $p_{1}$ | $R^{2}$ |  | |  |  |  | |  |
| 3692.4 | 0.3836 ± 0.0962 | **6.8512 × 10^-5^** | 0.4467 |  | |  |  |  | |  |
| **Interactions between handID and stimID** | | | | | | | | | | |
| **Model 1**: treFre ~ 1 + $k_{1}h\mathrm{andID}$+ $k_{2}\mathrm{stimID}$ + $k_{inter}\mathrm{stimID}*h\mathrm{andID}+$ 1\|subID + (-1 + stimID\|subID) + (-1 + handID\|subID) | | | | | | | | | | |
| AIC | $k_{1}$ | $p_{1}$ | $k_{2}$ | $p_{2}$ | | $k_{inter}$ | $p_{inter}$ | $R^{2}$ | | |
| 4200.9 | 0.216 ± 0.1865 | 0.2470 | 0.5915 ± 0.1311 | **6.7167 × 10^-6^** | | -0.2094 ± 0.0483 | **1.482 × 10^-6^** | 0.7581 | | |
| **Model 2**: trePow ~ 1 + $k_{1}h\mathrm{andID}$+ $k_{2}\mathrm{stimID}$ + $k_{inter}\mathrm{stimID}*h\mathrm{andID}+$ 1\|subID + (-1 + stimID\|subID) + (-1 + handID\|subID) | | | | | | | | | | |
| AIC | $k_{1}$ | $p_{1}$ | $k_{2}$ | $p_{2}$ | | $k_{inter}$ | $p_{inter}$ | $R^{2}$ | | |
| 13964 | -2.5158 ± 0.9335 | **0.0071** | -4.1523 ± 1.117 | **0.0002** | | 1.3305 ± 0.3335 | **6.8154 × 10^-5^** | 0.5414 | | |
| **Model 3**: treAmpInst ~ 1 + $k_{1}h\mathrm{andID}$+ $k_{2}\mathrm{stimID}$ + $k_{inter}\mathrm{stimID}*h\mathrm{andID}+$ 1\|subID + (-1 + stimID\|subID) + (-1 + handID\|subID) | | | | | | | | | | |
| AIC | $k_{1}$ | $p_{1}$ | $k_{2}$ | $p_{2}$ | | $k_{inter}$ | $p_{inter}$ | $R^{2}$ | | |
| 2034.7 | 0.1362 ± 0.0484 | **0.0050** | 0.1885 ± 0.0774 | **0.0149** | | -0.0668 ± 0.0315 | **0.0341** | 0.3038 | | |
| **Model 4**: treFreInst ~ 1 + $k_{1}h\mathrm{andID}$+ $k_{2}\mathrm{stimID}$ + $k_{inter}\mathrm{stimID}*h\mathrm{andID}+$ 1\|subID + (-1 + stimID\|subID) + (-1 + handID\|subID) | | | | | | | | | | |
| AIC | $k_{1}$ | $p_{1}$ | $k_{2}$ | $p_{2}$ | | $k_{inter}$ | $p_{inter}$ | $R^{2}$ | | |
| 3793.1 | 0.208 ± 0.1043 | 0.0462 | 0.4054 ± 0.1056 | **0.0001** | | -0.0841 ± 0.0446 | 0.0597 | 0.4159 | | |
| **Interactions between handID and chronicID** | | | | | | | | | | |
| **Model 1**: treFre ~ 1 + $k_{1}h\mathrm{andID}$+ $k_{2}\mathrm{chronicID}$ + $k_{inter}\mathrm{chronicID}*h\mathrm{andID}+$ 1\|subID + (-1 + chronicID\|subID) + (-1 + handID\|subID) | | | | | | | | | | |
| AIC | $k_{1}$ | $p_{1}$ | $k_{2}$ | $p_{2}$ | | $k_{inter}$ | $p_{inter}$ | $R^{2}$ | | |
| 2345.7 | 0.2964 ± 0.2192 | 0.1765 | -1.3771 ± 0.7387 | 0.0625 | | -0.2704 ± 0.5043 | 0.5919 | 0.8122 | | |
| **Model 2**: trePow ~ 1 + $k_{1}h\mathrm{andID}$+ $k_{2}\mathrm{chronicID}$ + $k_{inter}\mathrm{chronicID}*h\mathrm{andID}+$ 1\|subID + (-1 + chronicID\|subID) + (-1 + handID\|subID) | | | | | | | | | | |
| AIC | $k_{1}$ | $p_{1}$ | $k_{2}$ | $p_{2}$ | | $k_{inter}$ | $p_{inter}$ | $R^{2}$ | | |
| 8364.3 | -2.6719 ± 1.0445 | 0.0106 | 4.4746 ± 3.2193 | 0.1648 | | 1.7921 ± 2.3816 | 0.4519 | 0.5862 | | |
| **Model 3**: treAmpInst ~ 1 + $k_{1}h\mathrm{andID}$+ $k_{2}\mathrm{chronicID}$ + $k_{inter}\mathrm{chronicID}*h\mathrm{andID}+$ 1\|subID + (-1 + chronicID\|subID) + (-1 + handID\|subID) | | | | | | | | | | |
| AIC | $k_{1}$ | $p_{1}$ | $k_{2}$ | $p_{2}$ | | $k_{inter}$ | $p_{inter}$ | $R^{2}$ | | |
| 1281.7 | 0.1237 ± 0.0720 | 0.0860 | -0.3154 ± 0.2045 | 0.1231 | | 0.0638 ± 0.1622 | 0.6942 | 0.3320 | | |
| **Model 4**: treFreInst ~ 1 + $k_{1}h\mathrm{andID}$+ $k_{2}\mathrm{chronicID}$ + $k_{inter}\mathrm{chronicID}*h\mathrm{andID}+$ 1\|subID + (-1 + chronicID\|subID) + (-1 + handID\|subID) | | | | | | | | | | |
| AIC | $k_{1}$ | $p_{1}$ | $k_{2}$ | $p_{2}$ | | $k_{inter}$ | $p_{inter}$ | $R^{2}$ | | |
| 2047.4 | 0.2923 ± 0.1175 | 0.0130 | -0.4723 ± 0.3078 | 0.1262 | | -0.1879 ± 0.2672 | 0.4819 | 0.4662 | | |

DBS=deep brain stimulation; stimID=stimulation condition index, 0 and 1 indicate no DBS and continuous DBS (cDBS), respectively; treFre=tremor frequency; handID=index for tremor pre-dominant hand (0) or the other hand (1); subID=subject index; AIC=Akaike information criterion; trePow=tremor power (%) normalized against the total power in a broad frequency band of 1-25 Hz; treAmpInst=cycle by cycle tremor amplitude instability; treFreInst= cycle by cycle tremor frequency instability; inter=interaction; chronicID= acute or chronic DBS index. In each model, an independent random slope(s) between the predictor(s) and the dependent variable as well as an independent random intercept (s) were included. For each GLME model, the parameters were estimated based on maximum likelihood using Laplace approximation, the AIC, estimate value with standard error of the coefficient (*k* ± *SE*), pre-corrected *p*-value (*p*), and proportion of variability in the response explained by the fitted model (𝑅^2^) were reported. P-values survive multiple comparison correction following FDR approach were highlighted.

**
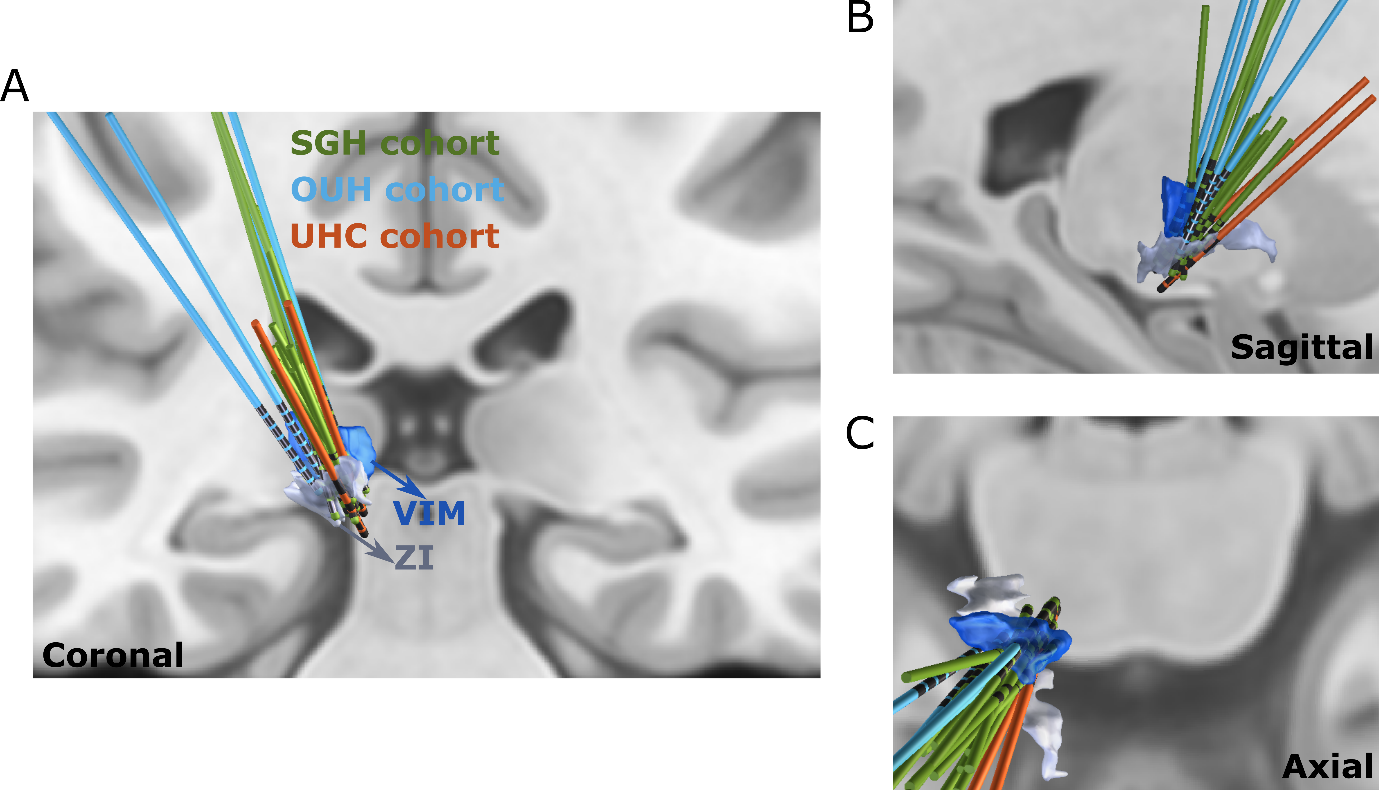
**

**Supplementary Figure 1. Lead placements.** 3D reconstruction in coronal (A), sagittal (B), and axial (C) views of all analysed DBS leads (except P10 whose scans are not available) localized in standard Montreal Neurological Institute (MNI)-152_2009b space using Lead-DBS MATLAB toolbox (version 2.6.0) (Horn et al., 2019). Electrodes in the left hemisphere were mirrored to the right hemisphere. SGH = St George’s Hospital; OUH = Oxford University Hospital; UHC = University Hospital Cologne.

**
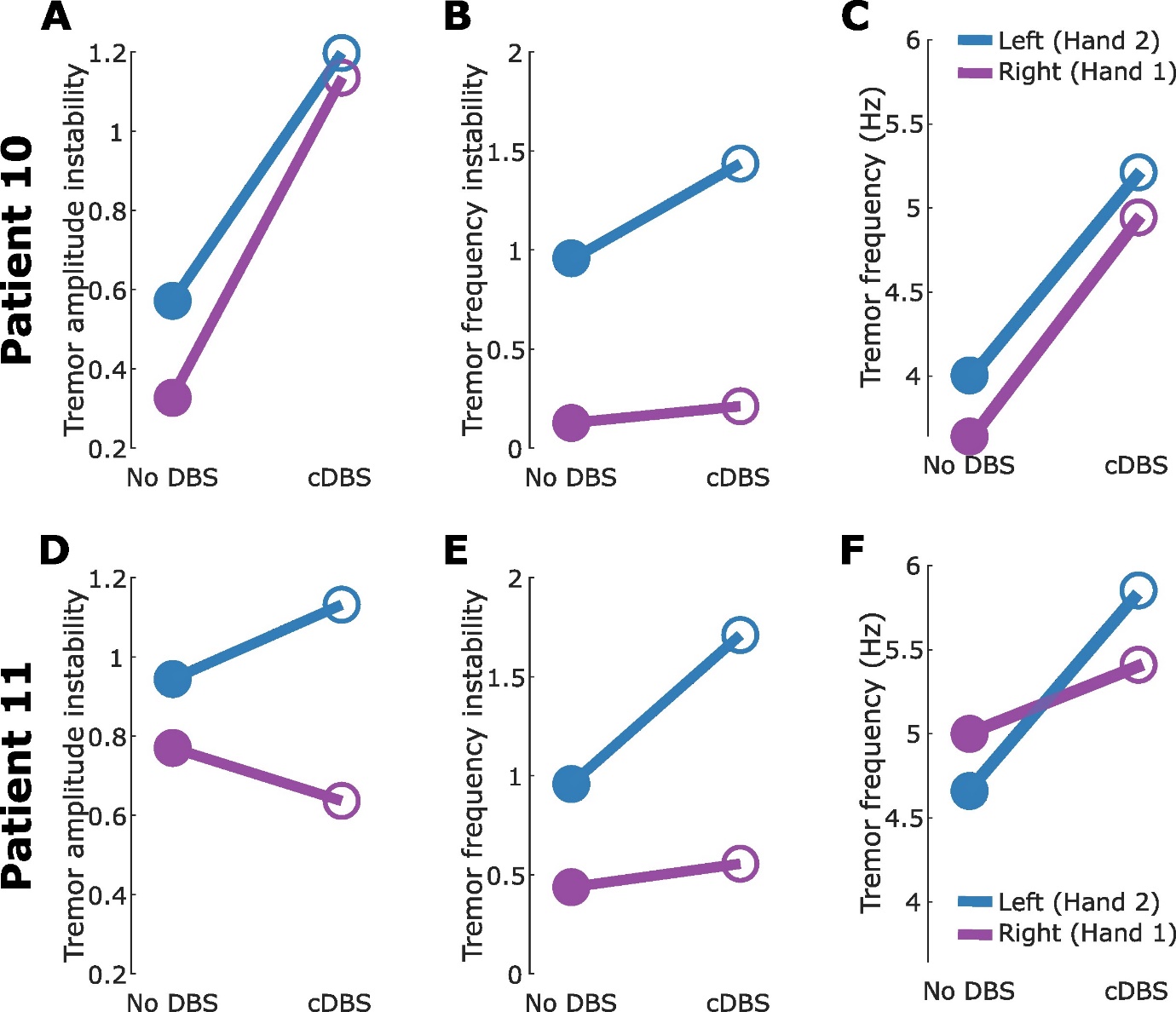
**

**Supplementary Figure 2. Tremor characteristics for those two patients participated in the phase-locked DBS experiment**. (A)-(B) Tremor amplitude (A) and frequency (B) instability during no DBS and cDBS for left (blue) and right (purple) hands for patient 10. (C) Tremor frequency during no DBS and cDBS for left and right hands. (D)-(F) The same as (A)-(D) but for patient 11. Please note that for both patients, the tremor was more pronounced in the right hand.

**References**

1. He S, Baig F, Mostofi A, Pogosyan A, Debarros J, Green AL, Aziz TZ, Pereira E, Brown P, Tan H. Closed‐Loop Deep Brain Stimulation for Essential Tremor Based on Thalamic Local Field Potentials. Movement Disorders. 2021 Apr;36(4):863-73.
2. He S, West TO, Plazas FR, Wehmeyer L, Pogosyan A, Deli A, Wiest C, Herz DM, Simpson T, Andrade P, Baig F. Cortico-thalamic tremor circuits and their associations with deep brain stimulation effects in essential tremor. Brain. 2024 Nov 27:awae387.
3. Horn A, Li N, Dembek TA, et al. Lead-DBS v2: towards a comprehensive pipeline for deep brain stimulation imaging. Neuroimage. 2019;184:293-316.
